# Reversible strain control of magnetic anisotropy in magnetoelectric heterostructures at room temperature

**DOI:** 10.1038/srep37429

**Published:** 2016-11-21

**Authors:** Margo Staruch, Daniel B. Gopman, Yury L. Iunin, Robert D. Shull, Shu Fan Cheng, Konrad Bussmann, Peter Finkel

**Affiliations:** 1 Materials Science and Technology Division, Naval Research Laboratory, Washington, DC 20375, USA; 2 Materials Science and Engineering Division, National Institute of Standards and Technology, Gaithersburg, MD, 20899-8552, USA; 3 Institute of Solid State Physics, Russian Academy of Science, Chernogolovka, 142432, Russia

## Abstract

The ability to tune both magnetic and electric properties in magnetoelectric (ME) composite heterostructures is crucial for multiple transduction applications including energy harvesting or magnetic field sensing, or other transduction devices. While large ME coupling achieved through interfacial strain-induced rotation of magnetic anisotropy in magnetostrictive/piezoelectric multiferroic heterostructures has been demonstrated, there are presently certain restrictions for achieving a full control of magnetism in an extensive operational dynamic range, limiting practical realization of this effect. Here, we demonstrate the possibility of generating substantial reversible anisotropy changes through induced interfacial strains driven by applied electric fields in magnetostrictive thin films deposited on (0 1 1)-oriented domain-engineered ternary relaxor ferroelectric single crystals with extended temperature and voltage ranges as compared to binary relaxors. We show, through a combination of angular magnetization and magneto-optical domain imaging measurements, that a 90° in-plane rotation of the magnetic anisotropy and propagation of magnetic domains with low applied electric fields under zero electric field bias are realized. To our knowledge, the present value attained for converse magnetoelectric coupling coefficient is the highest achieved in the linear piezoelectric regime and expected to be stable for a wide temperature range, thus representing a step towards practical ME transduction devices.

Magnetoelectric (ME) composite heterostructures have opened the door to practical devices allowing for voltage tuning of magnetism, which has led to breakthroughs in applications such as non-volatile memory, energy harvesting, magnetic field sensing, and potentially many other devices^1^,^2^,^3^. Demonstrated multifunctionality of ME materials consisting of piezoelectric and magnetostrictive components, where an applied electric field results in a change in magnetic properties, can be attributed to an interfacial strain mediated coupling between the piezoelectric/magnetostrictive components^4^. Thus for optimal device performance, both the piezoelectric and piezomagnetic coefficients (*d*^*e*^_*ij*_ and *d*^*m*^_*kl*_ respectively) as well as the strain transfer at the interface must be maximized. However, current performance of ME composites is either limited by ME coupling that is only optimized in a narrow range of temperatures or at large electric field bias, or due to bulk fabrication processes that limit coupling at the interface. To provide a path towards real-world devices, it is critical to boost non-resonant ME coupling while operating both at room temperature and in the linear piezoelectric regime.

Previous studies of ME composites generally utilize high electric field (E ≥ 1 MV/m), and thus electrostrictive control of magnetism, or a phase transition that is limited to a narrow temperature range to achieve large converse magnetoelectric (CME) coupling^5^,^6^,^7^. In particular, relaxor ferroelectrics are widely used in ME composites due to extremely high piezoelectric coefficients several times larger than those of lead zirconium titanate (PZT) based ceramics^8^,^9^,^10^,^11^. However, there has been little focus on ternary Pb(In_1/2_Nb_1/2_)O_3_-Pb(Mg_1/3_Nb_2/3_)O_3_-PbTiO_3_ (PIN-PMN-PT) single crystals that have d_33_ values similar to those of the binary systems PMN-PT or Pb(Zn_1/3_Nb_2/3_)O_3_-PbTiO_3_ (PZN-PT) but with a higher rhombohedral to tetragonal transition temperature. As a consequence, the PIN-PMN-PT crystals have a larger useable temperature range, as well as higher coercive electric fields (*E*_*C*_) compared to other relaxor ferroelectrics^12^,^13^.

The figure of merit for the CME effect, the coupling coefficient 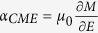 (where *μ*_0_ is the vacuum permeability and *M* is the magnetization), may suggests that one path towards improving the ME coupling is by engineering the magnetic material to provide the largest value of Δ*M,* which could be achieved through the use of single domain structures^14^,^15^ or other stratagems resulting a complete 90 degree rotation of the magnetization. A large value of saturation magnetization and high magnetostriction would also be expected to extend dynamic range and increase tenability of ME effective coupling, which is especially important for magnetic sensor and energy harvesting applications. Fe_50_Co_50_ is a well-known magnetostrictive material and also a promising candidate component in magnetic tunnel junctions^16^,^17^, which exhibits high permeability, reasonable saturation magnetostriction of ~65 ppm and the highest saturation magnetization of 1950 kA/m for Fe-based alloys^18^,^19^. As-sputtered FeCo films, unfortunately, generally possess very high coercive fields (*μ*_*0*_*H*_*C*_ > 10 mT) which leads to high hysteretic losses and a large bias field needed for optimal performance. Several approaches to softening this material have been identified including thermal annealing, depositing an underlayer, or including the FeCo in a multilayer heterostructured film^18^,^20^,^21^,^22^. An additional advantage for alternating layers of FeCo with a second (ferromagnetic or non-magnetic) material, such as Ag^23^,^24^, is that the saturation magnetostriction can be enhanced while reducing the value of *H*_*C*_.

The high increase in *E*_*C*_ in PIN-PMN-PT also extends the linear piezoelectric regime that, along with high *d*^*e*^_*ij*_, would enable larger and completely reversible Δ*M* changes without polarization switching and its corresponding losses, with the additional benefit of reducing effects of fatigue and aging that may alter the ME behavior of relaxor piezocrystals in composites^25^. Herein, by using thin films of FeCo and FeCo/Ag multilayers deposited on (0 1 1) cut PIN-PMN-PT single crystals we show that magnetization in these architectures can be rotated by 90° under low applied electric field at room temperature resulting in one of the highest values of α_CME_ reported thus far.

## Results

### Magnetization rotation with *E* < *E*_*C*_

**Figure 1:**
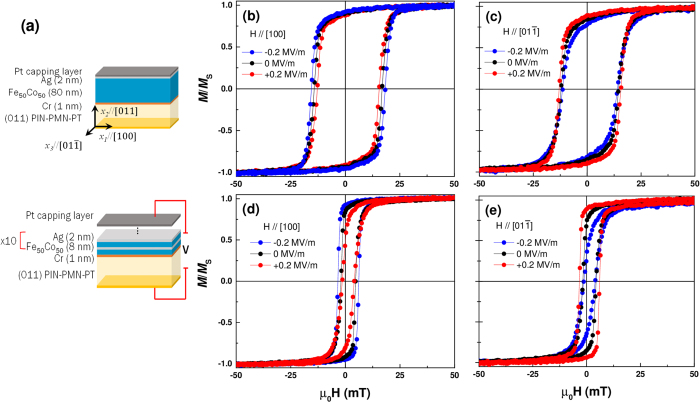
A schematic of (**a**) a FeCo film and a FeCo/Ag multilayer film on (011) oriented PIN-PMN-PT substrates. Room temperature in-plane magnetic hysteresis loops (**b**,**c**) for FeCo with magnetic field respectively parallel to the [100] and [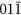] axes of PIN-PMN-PT, and (**d**,**e**) for the FeCo/Ag multilayer with magnetic field respectively parallel to the [100] and [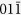] directions.

The configurations of the films and the single crystal substrate are given in Fig. 1(a). To investigate the effects of layering on the magnetic properties of the FeCo, magnetic field dependent magnetization loops (*M* vs. *μ*_*0*_*H*) were taken with a vibrating sample magnetometer (VSM) at 300 K along the [1 0 0] and [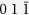] in-plane directions as shown in Fig. 1(b–e), as well as normal to the film (Figure S2). The saturation magnetization (*M*_*S*_) was determined to be 1171.3 ± 70 kA/m and 1091.2 ± 66 kA/m for the FeCo and FeCo/Ag films, respectively. Similar to previous reports for as-deposited FeCo films, a large coercive field of 15.5 mT (12.3 ± 0.5 kA/m) was observed in-plane. However, with the addition of 2 nm Ag layers *H*_*C*_ decreased to 3.4 ± 0.3 mT (2.7 ± 0.5 kA/m). It is likely that the introduction of Ag played a role in restricting the FeCo grain size consistent with previous studies that demonstrated a difference in size of the polycrystalline grains with film thickness between 5 nm and >10 nm. In accordance with Hoffmann’s ripple theory the decreased grain size would decrease the film’s coercivity^18^,^21^. For both films, the value of the remanent magnetization (*M*_*R*_) normalized to saturation (*M*_*S*_) is slightly higher for hysteresis loops measured along [1 0 0] as compared to [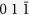], but does not definitively point toward any strong uniaxial anisotropy. However, the out-of-plane remanent magnetization was an order of magnitude smaller than after saturating the magnetization in either of the two in-plane directions, suggesting that the magnetic easy axis lies in-plane for both FeCo and FeCo/Ag consistent with previous reports for films with similar thickness^23^.

A positive electric field, corresponding to a negative electrical potential difference between the magnetic multilayer and the backside of the 4 mm thick PIN-PMN-PT substrate, induces a compressive strain along the [1 0 0] direction and a smaller tensile strain along the [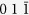] axis. A negative electric field will swap the direction of the strain along the two axes. These opposite strains should cooperatively rotate the magnetic easy axis in a manner consistent with magnetoelastic theory, minimizing the magnetoelastic energy as given by *E* = −3/2*λσ*cos^2^*θ*, where *σ* is the applied stress and *θ* the angle between the magnetization and stress. For positive λ as is found for FeCo and FeCo/Ag films, the easy direction of magnetization will tend to align in the direction of tensile stress. Potentially, the observed large anisotropic piezostrain could also enhance the in-plane rotation of the magnetization as compared to an isotropic biaxial strain imposed on a film from (0 0 1) oriented crystals^26^. The changes in the magnetic hysteresis loops with ±0.2 MV/m applied electric field (much lower than *E*_*C*_) for the pure FeCo film are visible in Fig. 1. The main effect is a slight decrease in the *M*_*R*_/*M*_*S*_ ratio and coercivity when either a positive *E* is applied and *H* is parallel to [1 0 0] or a negative *E* is applied with *H* parallel to [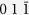]. However, the effects of electric field are much more evident in the FeCo/Ag multilayer film. A much larger change in *M*_*R*_ suggests an enhanced ME effect as compared to the FeCo film and implies that the magnetic easy axis rotates from [1 0 0] towards the [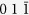] in-plane direction. When the direction of the field is switched, the magnetic easy axis is rotated further towards the [1 0 0] direction with a negative electric field and rotated towards 90° in plane with a positive electric field. The softer FeCo/Ag multilayer film shows a much higher change in anisotropy with applied electric field than the FeCo, possibly due to the multilayer’s higher value of *λ*_s_^23^,^24^.

**Figure 2:**
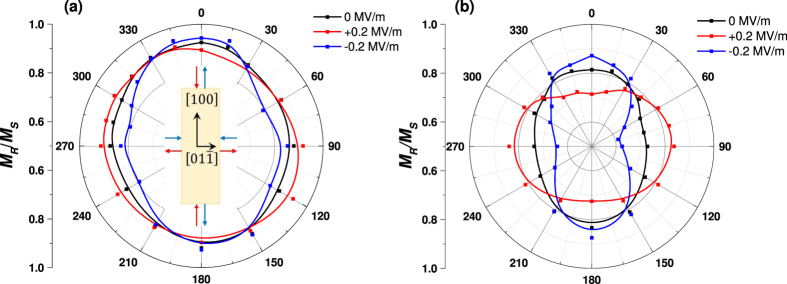
The in-plane angular dependence of the remanent magnetization (normalized to saturation) at zero electric field and ±0.2 MV m^−1^ for (**a**) a FeCo film and (**b**) a FeCo/Ag multilayer film. The direction of strain imposed by the substrate along the two in-plane axes is shown in the inset.

To clearly depict the effects of electric field for both samples, the angular dependence of *M*_*R*_/*M*_*S*_ at 0 MV/m and ±0.2 MV/m is shown in Fig. 2. The values don’t change significantly with rotation for the FeCo film in zero electric field, but there is a slight maximum at ±[1 0 0] and a slight change is noted with application of electric field ***E*** > 0. With ***E*** = −0.2 MV/m, the development of local maxima along the ±[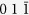] directions with uniaxial tensile strain is observed. A four-fold anisotropy is characteristic of bcc-FeCo and has been previously observed in epitaxial films^27^,^28^, however other contributions (e.g., surface roughness resulting from the polishing process of the PIN-PMN-PT crystal) may also influence the anisotropy energy in the present film. For the FeCo/Ag film at ***E*** = 0, although the *M*_*R*_/*M*_*S*_ ratio is slightly decreased as compared to the FeCo film, the angular dependence shows a more well defined easy axis along the [1 0 0] direction, and that result may be related to the relatively high uniaxial anisotropy energy *K*_u_ previously observed in FeCo/Ag films^29^. The effects of an applied electric field on the magnetization is also much more obvious in the multilayered film. Upon application of a negative electric field, a clear uniaxial anisotropy emerges along the [1 0 0] direction in the multilayer sample, which is consistent with the development of a large tensile stress. Application of a positive 0.2 MV/m electric field is accompanied by a distinct 90° rotation of the zero field curve as a result of a rotation of the anisotropy axes. It is envisaged that with even higher applied electric fields, a clearer uniaxial anisotropy along [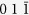] would be developed and accordingly the ME effect enhanced. The results for the present FeCo and FeCo/Ag multilayer films are reminiscent of a previously observed change in anisotropy from easy-plane to uniaxial for pure FeCo films grown directly on a substrate having a Cu underlayer^30^.

**Figure 3:**
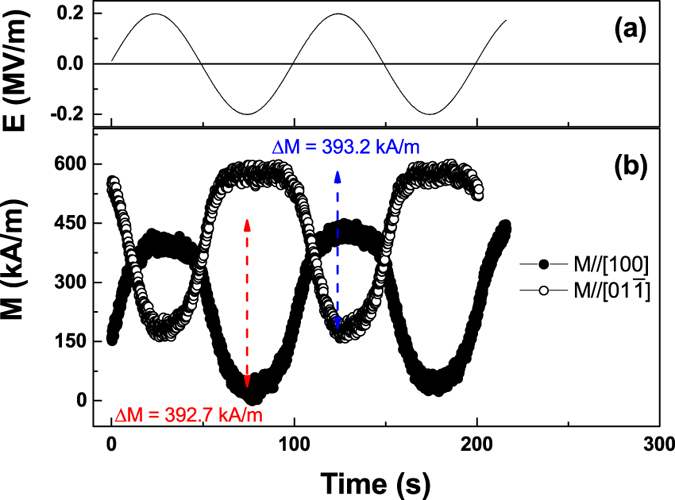
(**a**) The time dependent applied electric field and (**b**) the resultant change in magnetization of the FeCo/Ag multilayered film measured along [100] and [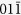] in-plane directions of the PIN-PMN-PT crystal.

The nearly complete 90° rotation of the easy axis in the FeCo/Ag multilayer film mentioned above also becomes evident if we compare the change in magnetization along the [1 0 0] and [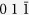] directions at 0.5 mT (0.4 kA/m) bias field with an AC electric field (as shown in Fig. 3) measured in a VSM. Remarkably, the values of Δ*M* between ± 0.2 MV/m were determined to be 392.7 ± 48.1 kA/m and 393.2 ± 48.1 kA/m along [1 0 0] and [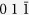], respectively. The rotation of the magnetization appears to be predominantly constrained in the plane of the film with very little rotating parallel to the film normal. This is consistent with the hysteresis loops suggesting that [0 1 1] is a hard axis in the present films. The observed change in magnetization corresponds to a α_CME_ of 1.23 ± 0.15 × 10^−6^ s/m, which is among the highest values of CME coupling reported thus far^5^,^31^,^32^,^33^. It should be noted that this value of the coupling coefficient is determined far from mechanical resonance that would be expected to inflate this value, and does not rely on 180° ferroelectric polarization switching that is expected to fatigue the sample and provide a nonlinear magnetization response.

Interestingly, the sign and magnitude of the magnetic bias field used for the measurements has consequences for the stability of the ME effect. Measurements at zero magnetic field bias for both samples resulted in an extremely large but irreversible change in magnetization (See Supplementary Information, Figure S3). Addition of a uniaxial strain at zero magnetic bias could rotate the magnetization towards either one of the two equivalent directions for the magnetization, *i.e.* ±[1 0 0] for negative electric field and ±[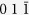] for positive electric fields. Consequently, the sign of the magnetization can be switched with an AC electric field. Similar CME behavior is also observed when the sample is brought down from saturation with positive magnetic field and the electric field is cycled while maintaining a negative magnetic field bias near *H*_*C*_, with the magnetization irreversibly switching to the −[1 0 0] direction.

**Figure 4:**
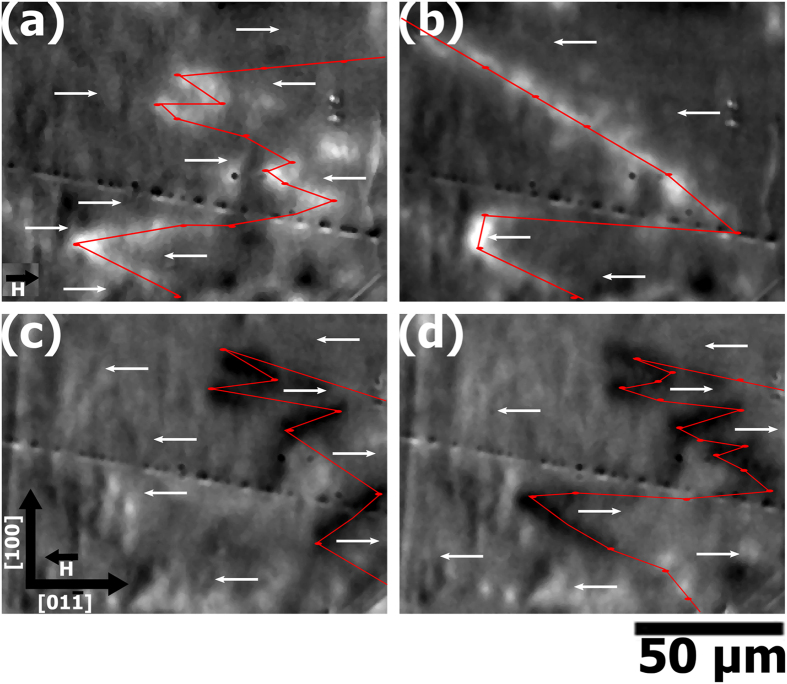
MOIF domain images of CoFe/Ag multilayers under applied magnetic and electric fields. (**a**) μ_0_*H* = 5.6 mT, E = 0; (**b**) μ_0_*H* = 5.6 mT, E = −0.2 MV/m. Subfigures (**c**,**d**) are domain images under the same magnitude applied magnetic and electric fields, but of opposite polarity (e.g., μ_0_*H* = −5.6 mT, E = 0 and μ_0_*H* = −5.6 mT, E = +0.2 MV/m respectively). Domain walls are highlighted with overlaid red line and ellipse markers, and inferred magnetic domain configurations are shown with white arrows.

We also investigate the electric-field induced magnetic domain transformation using a magneto-optic indicator film (MOIF) technique to observe the domain configurations under applied electric and magnetic fields. Using a full-field polarizing microscope, we observe local regions of the MOIF film that are tilted out-of-plane by stray flux emerging from domain walls (or edges) in the FeCo/Ag multilayered film. We can then infer the magnetic domain configurations in the FeCo/Ag multilayers based upon information about the domain walls. Figure 4 presents changes to the MOIF contrast (domain walls with overlaid red line and ellipse markers) and inferred magnetic domain configurations (white arrows) induced by strain-mediated magnetoelectric coupling. For both positive and negative magnetic field values, domain formation in the FeCo/Ag multilayered film on PIN-PMN-PT is evident by the bright and dark lines in Fig. 4(a,c) respectively. Figure 4(b) shows the effect of applying E = −0.2 MV/m. Electric-field induced domain wall motion is evident in the appearance of a domain wall (bright contrast) stretching diagonally across the top two-thirds of the figure. Additional evidence can be found in the flattening (along the [100] direction) of the curved domain structure in the bottom third of the figure. This behavior is similarly captured in Fig. 4(c,d) showing the effect of applying an electric field of +0.2 MV/m in the presence of an applied magnetic field of the opposite polarity (yielding dark contrast at the domain walls). Similarly, Fig. 4(b,d) also shows the propagation of domain walls from right-to-left after turning on the electric field clearly demonstrating that electric field induced domain wall motion contributes to the observed change in bulk magnetic properties.

### Role of strain from the piezocrystal

**Figure 5:**
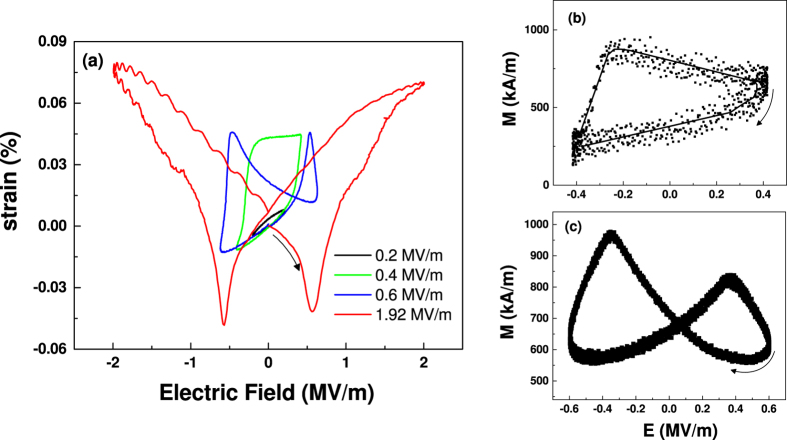
(**a**) Strain-electric field curves with displacement and field along the [011] direction for the PIN-PMN-PT crystal. Corresponding magneto-electric curves for (**b**) ±0.4 MV/m and (**c**) ±0.6 MV/m applied electric field cycles at 3.5 and 1 mT, respectively. Arrows indicate the direction of the curve with applied AC field.

We have so far only been examining the reversible voltage tuning of magnetization by measuring only in the linear piezoelectric regime of the present heterostructures. With larger applied AC electric fields, the electrically-induced strain in PIN-PMN-PT (strain measured along [0 1 1]) shows nonlinear behavior for several minor loops as shown in Fig. 5, measured with a linear variable differential transformer and a Sawyer-Tower setup. At 0.6 MV/m, two large peaks in strain [Fig. 5(a)] are observed corresponding to the 180° switching of the polarization near *E*_*C*_. Measuring the resultant change in the magnetization for the FeCo/Ag film [Fig. 5(c)] shows a nearly identical butterfly loop, confirming that strain coupling at the interface is driving the converse ME effect. Addition of the Cr underlayer should ensure that modulation of charge is decoupled from the magnetic layer, and so there are no contributions from charge mediated ME coupling. Hence, the peaks in magnetization in Fig. 5(c) are a pure strain effect^34^. When an intermediate 0.4 MV/m field is applied, non-180° polarization rotation results in large hysteresis with significant remanent strain. These two bistable strain states are separated by about 500 ppm that translates to two stable values of the magnetization as revealed in Fig. 5(b), which could be useful in memory devices to write up and down states. Similar to the previously observed stable and tunable remanent strain states in PMN-PT^35^, tunable remanent magnetization with Δ*M* greater than 46% between stable states can be achieved with relatively low electric field (<*E*_*C*_) and could be increased through further exploration of the piezostrain in the PIN-PMN-PT crystal.

### Enhancement of ME coupling

**Figure 6:**
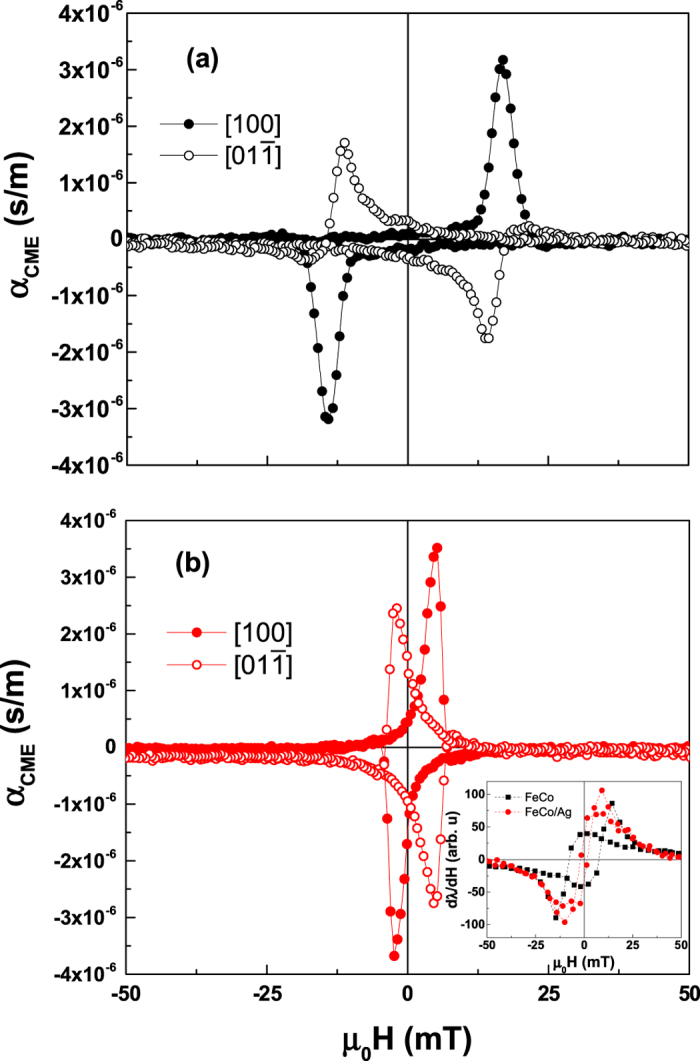
The converse magnetoelectric coupling coefficient α_CME_ as calculated from data shown in Fig. 1 for (**a**) FeCo and (**b**) FeCo/Ag multilayer films on PIN-PMN-PT. The inset shows the piezomagnetic coefficient (d*λ*/d*H*) as calculated from magnetostrictive measurements.

Finally, after examining the importance of magnetic and electric fields, the CME coupling coefficient was evaluated at many values of *H* from calculating Δ*M* between ±0.2 MV/m from the *M* vs. *H* loops in Fig. 1(b–e) and the results are presented in Fig. 6. Ultimately, the maximum achievable values of converse magnetoelectric coupling for both the FeCo and FeCo/Ag film are nearly identical at bias fields near *H*_*C*_ for *H* parallel to the [1 0 0] direction, with the maximum value for FeCo/Ag of 3.5 × 10^−6^ s/m. This value represents a more than 10% increase compared to that for pure FeCo. Notably, α_CME_ could be expanded as 
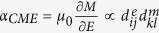
 36,37 where 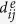 and 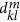 are the piezoelectric and piezomagnetic coefficients, respectively. From magnetostriction measurements, the piezomagnetic coefficient *d*^m^ = *dλ*/*dH* was evaluated (shown in the inset of Fig. 6). For the present samples, the experimentally observed increase of *d*^m^ by ~15% is consistent with previous reports^23^ and is in line with the modest difference in α_CME_. This suggests that the strain coupling in the material is not significantly influenced by the addition of Ag layers. Again, we would like to stress that this value of 3.5 × 10^−6^ s/m is 5 orders of magnitude larger than α_CME_ found for single phase multiferroics and one of the highest reported for heterostructured composites to our knowledge in the literature (comparison shown in Table 1). We attribute this sizable multiferroic coupling to the sum of combined effects including a principally uniaxial in-plane strain and an enhanced piezomagnetic coefficient in the Fe_50_Co_50_/Ag multilayer film. Furthermore, for the present FeCo/Ag multilayer on PIN-PMN-PT, we demonstrate giant non-resonant ME coupling at room temperature and at applied fields below the electric coercive field (accordingly not utilizing electrostriction and not electrically fatiguing the crystal), a benchmark which distinguishes itself in comparison to previous studies and thus represents a significant step towards energy efficient, fatigue resistant ME transduction devices.

## Conclusion

In summary, Fe_50_Co_50_ and Fe_50_Co_50_/Ag multilayered films were deposited on (0 1 1) cut and poled PIN-PMN-PT single crystals allowing for reversible voltage control of magnetism with a broader operational range. Generating a laminated CoFe multilayer with thin Ag spacers significantly reduced the coercive field and favored a uniaxial anisotropy that showed 90° in-plane rotation of the magnetization with strain imposed by the substrate. We have demonstrated tuning of magnetization and direct observation of propagation of magnetic domains as an immediate result of the large piezoelectric strain response of PIN-PMN-PT. The maximum value of converse magnetoelectric coupling coefficient in the linear regime was revealed to be at least 3.5 × 10^−6^ s/m for the FeCo/Ag multilayer film on PIN-PMN-PT. The 10% increase in this value as compared to pure FeCo is primarily due to an enhancement of the piezomagnetic coefficient in the multilayer film. The increased α_CME_ and lowered bias field needed for optimal performance due to decreased *H*_*C*_ consequently make the present strain-mediated heterostructural composite with the FeCo/Ag film a promising candidate for multiferroic devices.

### Experimental Information

#### Sample Fabrication

Rhombohedral PIN-PMN-PT single crystals with composition near a morphotropic phase boundary were grown by a modified Bridgman method (provided by TRS Technologies^[33a]^). One of the (011) surfaces of the single crystals was mechanically polished down to ~40 nm roughness before sputter deposition of thin films of the ferromagnetic material. A 1 nm Cr adhesion layer was first sputtered on the polished substrate with an AJA magnetron sputtering system^[33a]^, followed by either 80 nm of Fe_50_Co_50_ (FeCo) or a multilayer consisting of 8 nm FeCo and 2 nm Ag repeated 10 times, and completed with a Pt capping layer. The pure FeCo film was also topped with Ag before the Pt capping layer was deposited as shown in Fig. 1(a). Identical films were also grown on Kapton and the displacement measured with a capacitance gauge was used for calculation of the magnetostriction (Figure S1)^38^.

#### Magnetoelectric Characterization

A LakeShore vibrating sample magnetometer (VSM)^[33a]^ was used to characterize the magnetic hysteresis loops of the samples. Using this setup with electrodes wired to the sample, *in-situ* magnetic measurements were performed with either a DC or AC electric field applied to the PIN-PMN-PT sample. Strain vs. electric field measurements were done using a custom Sawyer-Tower measurement system with a linear variable differential transformer.

#### Magnetic Domain Imaging

Magnetic domain observations were carried out using the magneto-optic indicator film (MOIF) technique^39^. After applying a saturating field to the film, we reduced the applied magnetic field, *H*, (along the [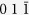] direction) to zero, reversed its direction and increased it to a finite value of 4.5 ± 0.2 kA/m (*μ*_*0*_*H* = 5.6 ± 0.2 mT) which is just below the coercivity of the thin film. The indicator film’s in-plane magnetization can be rotated out of the plane in places where there is flux leakage from the sample, for example at a domain wall or at an edge of the sample. As a consequence, light incident upon the indicator film experiences a large magneto-optic Faraday rotation where the film is magnetized perpendicular to the plane. From the polarization contrast at these locations, an image of domain walls in the sample can be generated.

## Additional Information

**How to cite this article**: Staruch, M. *et al.* Reversible strain control of magnetic anisotropy in magnetoelectric heterostructures at room temperature. *Sci. Rep.* **6**, 37429; doi: 10.1038/srep37429 (2016).

**Publisher’s note:** Springer Nature remains neutral with regard to jurisdictional claims in published maps and institutional affiliations.

## Supplementary Material

Supplementary Information

## Figures and Tables

**Table 1:** Comparison of converse magnetoelectric coupling coefficient α_CME_ for several single phase and composite materials.

Multiferroic system	α_CME_ (s/m)	T (K)	ref.
TbMn_2_O_5_	2.1 × 10^−11^	3 K	40
Co_0.9_Fe_0.1_/Cu/Co_0.9_Fe_0.1_ on BiFeO_3_	1 × 10^−7^	300 K	41
La_0.67_Sr_0.33_MnO_3_ on BaTiO_3_	2.3 × 10^−7^	180 K	6
Co_40_Fe_40_B_20_ on PMN-PT	2 × 10^−6^	300 K	7
**Fe**_**50**_**Co**_**50**_**/Ag on PIN-PMN-PT**	**3.5 × 10**^**−6**^	**300 K**	***present work***
FeRh on BaTiO_3_	1.6 × 10^−5^	360 K	5
