# Supplementary material for: Reversible strain control of magnetic anisotropy in magnetoelectric heterostructures at room temperature
Source: Sci Rep. 2016 Nov 21;6:37429. doi: 10.1038/srep37429 (PMC5116636; doi:10.1038/srep37429)
Supplement: Supplementary Information [file srep37429-s1.pdf]

Supplementary information

Reversible strain control of magnetic anisotropy in magnetoelectric heterostructures at room temperature

Margo Staruch[1], Daniel B. Gopman[2], Yury L. Iunin[2,3], Robert D. Shull[2], Shu Fan Cheng[1], Konrad Bussmann[1], and Peter Finkel[1,]*

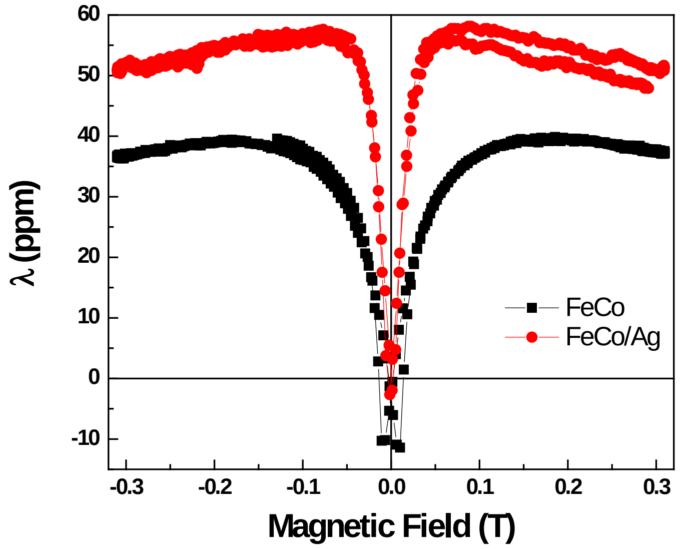

Figure S1. Magnetostriction measurements of FeCo and FeCo/Ag films deposited onto a 5 mil thick piece of Kapton. Magnetostriction was measured using the equations for magnetostrictive bimorphs developed in [1].

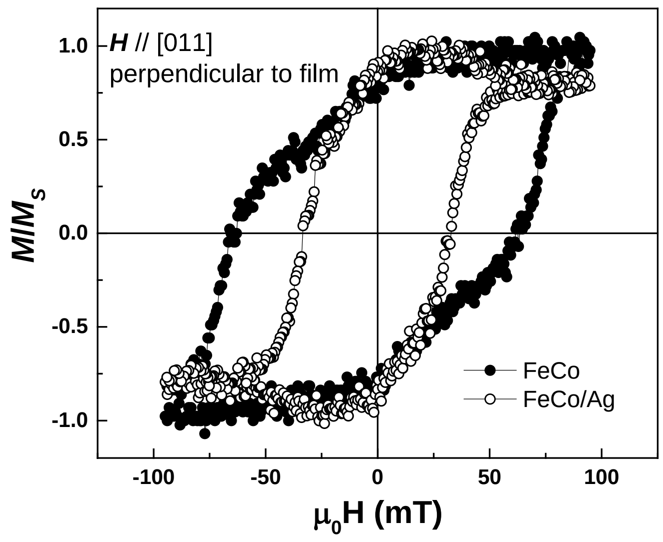

Figure S2. Magnetic hysteresis loops with magnetic field normal to the film (parallel to the [011] axis of the PIN-PMN-PT crystal).

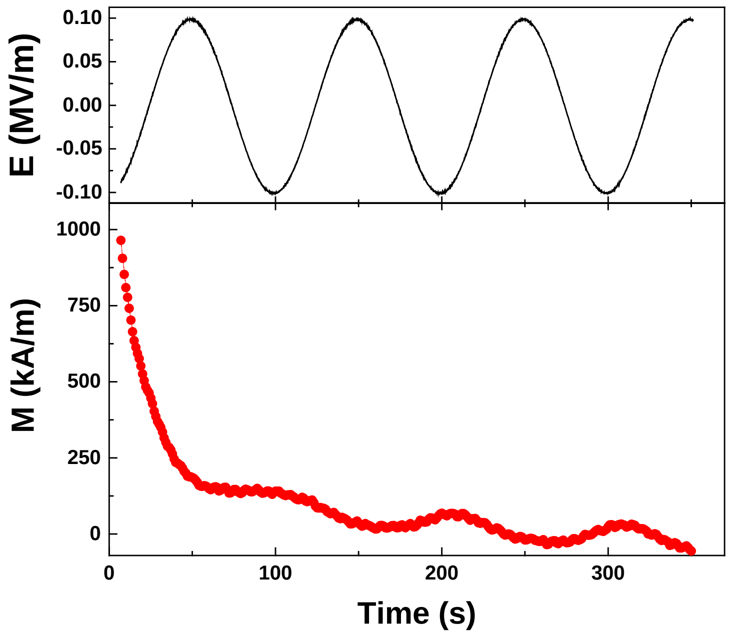

Figure S3. Magnetization of a FeCo/Ag film on PIN-PMN-PT measured with AC electric field at 0 mT magnetic field bias. A large but irreversible change in magnetization is observed in the time dependent data.

References

[1] du Trémolet de Lacheisserie, E. & Peuzin, J. C. 'Magnetostriction and internal stresses in thin films: the cantilever method revisited'. *J. Magn. Magn. Mater.* **152,** 231–232 (1996).
